# New cyclopentaquinoline hybrids with multifunctional capacities for the treatment of Alzheimer’s disease

**DOI:** 10.1080/14756366.2017.1406485

**Published:** 2017-12-06

**Authors:** Kamila Czarnecka, Małgorzata Girek, Karolina Maciejewska, Robert Skibiński, Jakub Jończyk, Marek Bajda, Jacek Kabziński, Przemysław Sołowiej, Ireneusz Majsterek, Paweł Szymański

**Affiliations:** a Department of Pharmaceutical Chemistry, Drug Analyses and Radiopharmacy, Faculty of Pharmacy, Medical University of Lodz, Lodz, Poland;; b Department of Medicinal Chemistry, Faculty of Pharmacy, Medical University of Lublin, Lublin, Poland;; c Department of Physicochemical Drug Analysis, Chair of Pharmaceutical Chemistry, Faculty of Pharmacy, Jagiellonian University Medical College, Krakow, Poland;; d Department of Clinical Chemistry and Biochemistry, Medical University of Lodz, Lodz, Poland

**Keywords:** Acetylcholinesterase inhibitors, Alzheimer’s disease, dementia, multifunctional drugs

## Abstract

Alzheimer’s disease (AD) is the most common progressive form of brain neurodegeneration and the most prevailing cause of dementia. Unfortunately, the aetiology of AD is not completely studied but different factors are associated with the development of AD such as among others low level of acetylcholine, aggregation of β-amyloid (Aβ), hyperphosphorylated tau protein, oxidative stress, and inflammation. The study encompass organic syntheses of 2,3-dihydro-1H-cyclopenta[b]quinoline with 5,6-dichloronicotinic acid and suitable linkers derivatives as multifunctional agents for AD treatment. Afterwards self-induced amyloid beta aggregation, inhibition studies of acetylcholinesterase and butyrylcholinesterase and molecular docking studies were performed. The results showed that **3b** compound exhibited the best acetylcholinesterase inhibitory activity, with IC_50_ value of 0.052 µM which is lower compared to references. Besides, all synthesised compounds showed good butyrylcholinesterase inhibitory activity with IC_50_ values from 0.071 to 0.797 µM. Compound **3b** exhibited strong Aβ_1–42_ aggregation inhibitory effect with 25.7% at 5 µM to 92.8% at 100 µM as well as good anti-inflammatory effect. Thus, new compounds could create new perspectives for further development as a multi-target-directed agent for AD treatment.

## Graphical Abstract

**Figure:**
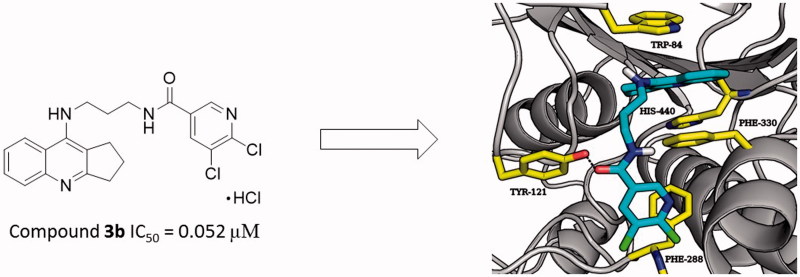

## Introduction

Alzheimer’s disease is a neurodegenerative, progressive disorder of central nervous system (CNS) that generally affects aged people. It is the most common cause of dementia which makes up 70% of senile dementia^1^. Today there are 47 million people living with dementia worldwide. By 2050 this number will have increased above 135 million^2^. The incidents of dementia rise with increasing age. Dementia affects 3.9 persons out of 1000 at the age ranging from 60 to 64^3^. Although the advanced age, the history of family with AD and heredity are the main risk factors. Researchers have identified another factors leading to AD, i.e. head injury, stroke, heart disease, hypertension, mutation of ApoE (ApoE4), hypercholesterolemia, diabetes mellitus type 2, insulin resistance, obesity, and smoking^4^ ^,^ ^5^. Clinically, AD is characterised by progressive decline of memory, learning processes, language, and personality changes which reduce the ability to basic activities of daily living. Histological and morphological brain changes in patients with AD is manifested by β-amyloids extracellular deposits, hyperphosphorylation tau protein and creation of neurofibrillary tangles inside neurons, impairment of cholinergic system, oxidative stress, and neuroinflammation including high levels of cytokines and resistin^6–8^. Abnormalities occur in cortical and subcortical grey matter especially in hippocampus, parahippocampal gyrus, thalamus, amygdala, entorhinal, and posterior cingulate cortex. The recent studies have showed that longitudinal white matter and white matter connected with limbic system networks are also impaired at patients with AD. In addition, the degeneration of white matter is not related with the degeneration of grey matter, and it occurs earlier. It creates the chance to earlier diagnosis before the first symptoms appear^9–11^. This structural and metabolic changes CNS are used to diagnose the purposes of AD including PET, MRI, DTI, and FA^12–14^. Supplementary diagnostic method tests intellectual function useful to determine the degree of mental impairment. Pathogenesis of AD is mighty intricate. Researches have suggested several hypotheses explaining pathomechanism of evolution of AD. There are: cholinergic hypothesis (cholinergic dysfunction), β-amyloid cascade (amyloid hypothesis), tau hyperphosphorylation, low concentration of steroids hormone, oxidative stress, and inflammation theory. Among them cholinergic hypothesis is well documented and has been globally validated. Abnormalities in central cholinergic neurotransmission are the most characteristic features connected with AD. The pathology of decreasing concentration of acetylcholine (ACh) in synaptic cleft is a low activity of choline acetyltransferase (ChAT), a specific enzyme that synthesises ACh from choline and acetate. The correlation between reduced activity of ChAT and severity of AD symptoms is scientifically proved. Besides ChAT acetylcholinesterase (AChE) and butyrylcholinesterase (BuChE) influence on cholinergic balance, which are two types of enzyme hydrolyse ACh. At healthy individuals, AChE has a crucial role in the regulation of the level of ACh while BuChE is less active. However, patients with AD who were observed with significant increase of BuChE activity (nonspecific esterase) correlated with decrease in AChE activity^4^ ^,^ ^15^ ^,^ ^16^. Food and Drug Administration (FDA) initially approved four acetylcholinesterase inhibitors acting on central cholinergic neurotransmission: tacrine, donepezil, galantamine, and rivastigmine. The first acetylcholinesterase reversible inhibitor approved by FDA against AD was tacrine. The mechanism of action tacrine is complicated and involves several pathways: cholinergic, gabaergic, glutaminergic, and nitrinergic. Nonetheless, the therapy with tacrine offers some benefits, serious side-effects including hepatotoxicity, gastrointestinal toxicity hypotension force FDA to withdraw tacrine from the market. Memantine was the last passed drug at anti-AD therapy and the first targeting the N-methyl-D-aspartate (NMDA) receptors and glutaminergic neurotransmission. Scientists proved that excess glutamate at synapses is associated with cytotoxicity and participates in pathological mechanism of AD. Currently, approved drugs provide only partial symptomatic relief and moderate as well as elevate daily living ability^4^ ^,^ ^5^ ^,^ ^17^. Complicated pathogenesis of AD requires new approaches to the drugs and therapy. The recent studies are focused to create the drug affecting on several pathways at the same time including cholinergic, glutaminergic, gabaergic, nitrinergic, and influence on β-amyloid and tau protein. This multi-target directed ligands (MTDLs) generate a new possibility of the therapy of AD. The MTDLs strategy is reflected by many new researches of tacrine derivatives. The purpose of the research is to increase activity and selectivity of derivatives, improve of pharmacokinetics, reduce side-effects of tacrine, and target on another pathomechanism. According to MTDLs theory, our researches are also focused on a development of new tetrahydroacridine derivatives as potential drugs against AD. From synthesised compounds containing multi-functional ligands and tacrine moiety, the most interesting are cholinesterase inhibitors creating double binding with catalytic anionic site (CAS), and peripheral anionic site (PAS) characterised also as inhibitors of Aβ^4^ ^,^ ^8^ ^,^ ^15–23^.

## Materials and methods

### Chemistry

All the chemical reagents used in the synthesis were obtained from commercial sources. ^1^H spectra were recorded on a Brucker Advance III 600 MHz spectrometer using DMSO-d6 or methanol-d4 as solvents. The chemical shifts were reported in parts per million (ppm), using tetramethylsilane (TMS) as an internal reference. High resolution mass spectra (HRMS) analysis was performed with the use of Agilent Accurate-Mass Q-TOF LC/MS G6520B system with dual electrospray (DESI) source (Agilent Technologies, Santa Clara, CA). The detector in the analysis was tuned in a positive mode with the use of Agilent ESI-L tuning mix in high resolution mode (4 GHz). The IR spectra were recorded in attenuated total reflectance (ATR) mode (Thermo Scientific Nicolet 6700, Thermo Electron Scientific Instruments Corporation, Madison, WI). Fourier transform was infrared spectrometer with smart ITR diamond adapter (Madison, WI). Conducted reactions were monitored by the thin layer chromatography (TLC). Anhydrous Na_2_SO_4_ was used to drying organic solutions. Melting points were determined using an Electrothermal apparatus with open capillaries and were uncorrected. The solvents were removed by rotary evaporation under reduced pressure. Flash chromatography – purifying technique, was performed using silica gel 60 (Merck). Intermediates **1a**–**1h** were prepared according to the previously literature method^24^.

### General procedure for the synthesis of compounds 2a–2h

A mixture of the tetrahydrofuran (THF) (8 ml), 2-chloro-4,6-dimethoxy-1,3,5-triazine (CDMT) (0.16–0.59 g, 0.91–3.36 mM), 5,6-dichloronicotinic acid (0.18–0.65 g, 0.9–3.4 mM) and dropwise of N-methylomorpholine (0.09–0.36 g, 0.91–3.36 mM) were added. The reaction was carried out about 2 h in the ice bath. Then (0.22–1.00 g, 0.91–3.36 mM) of proper diamine dissolved in THF (3 ml) was added to the mixture and the reaction has been continuing at room temperature for 24 h with stirring. Purification was performed by flash chromatography to give compounds **2a**–**2h** in good yield. Physical and spectral data are listed below.

#### 5,6-Dichloro-N-[2-(2,3-dihydro-1H-cyclopenta[b]quinolin-9-ylamino)-ethyl]-nicotinamide (2a)

Yield: 85%; beige solid; mp 175–178 °C; IR (KBr) ν (cm^−1^): 765.7; 1228.2; 1332.5; 1432.5; 1563.5; 2946.2; 3260.4; ^1^H NMR (600 MHz, CD_3_OD) δ 8.70 (1H, s, Ar), 8.35 (1H, d, *J* = 8.4 Hz, Ar), 8.32 (1H, s, Ar), 7.91 (1H, t, *J* = 8.1 Hz, Ar), 7.78 (1H, d, *J* = 8.4 Hz, Ar), 7.70 (1H, t, *J* = 7.9 Hz, Ar), 4.13 (2H, t, *J* = 6.0 Hz, CH_2_), 3.81 (2H, t, *J* = 5.9 Hz, CH_2_), 3.50–3.45 (2H, m, CH_2_), 3.25–3.19 (2H, m, CH_2_), 2.34 (2H, p, *J* = 7.8 Hz, CH_2_) (protons of NH groups invisible); MS (ESI) *m/z*: 400.1, 217.0, 185.1; MS-HR (ESI) calcd for C_20_H_18_Cl_2_N_4_O: 400.08577, found: 400.08768.

#### 5,6-Dichloro-N-[3-(2,3-dihydro-1H-cyclopenta[b]quinolin-9-ylamino)-propyl]-nicotinamide (2b)

Yield: 85%; beige solid; mp 131–133 °C; IR (KBr) ν (cm^−1^): 754.5; 1243.1; 1332.1; 1421.9; 1562.7; 2945.8; 3218.5; ^1^H NMR (600 MHz, CD_3_OD) δ 8.74 (1H, s, Ar), 8.39 (1H, d, *J* = 8.5 Hz, Ar) 8.34 (1H, s, Ar), 7.89 (1H, *t*, *J* = 7.7 Hz, Ar), 7.76 (1H, d, *J* = 8.8 Hz, Ar), 7.67 (1H, *t*, *J* = 7.8 Hz, Ar), 3.96 (2H, *t*, *J* = 6.7 Hz, CH_2_), 3.62 (2H, *t*, *J* = 6.6 Hz, CH_2_), 3.43 (2H, *t*, *J* = 7.3 Hz, CH_2_), 3.25–3.19 (2H, m, CH_2_), 2.32 (2H, p, *J* = 7.7 Hz, CH_2_), 2.10 (2H, p, *J* = 6.8 Hz, CH_2_) (protons of NH groups invisible); MS (ESI) *m/z*: 414.1, 231.0, 185.1; MS-HR (ESI) calcd for C_21_H_20_Cl_2_N_4_O: 414.10142, found: 414.10127.

#### 5,6-Dichloro-N-[4-(2,3-dihydro-1H-cyclopenta[b]quinolin-9-ylamino)-butyl]-nicotinamide (2c)

Yield: 78%; beige solid; mp 117–118 °C; IR (KBr) ν (cm^−1^): 758.1; 1227.8; 1363.6; 1417.8; 1556.1; 2938.9; 3258.9; ^1^H NMR (600 MHz, CD_3_OD) δ 8.68 (1H, s, Ar), 8.32–8.27 (2H, m, Ar), 7.83 (1H, *t*, *J* = 7.7 Hz, Ar), 7.73 (1H, d, *J* = 8.7 Hz, Ar), 7.61 (1H, t, *J* = 7.8 Hz, Ar), 3.87 (2H, *t*, *J* = 7.0 Hz, CH_2_), 3.48 (2H, *t*, *J* = 6.8 Hz, CH_2_), 3.43–3.37 (2H, m, CH_2_), 3.17 (2H, *t*, *J* = 7.9 Hz, CH_2_), 2.29 (2H, p, *J* = 7.8 Hz, CH_2_), 1.89–1.78 (4H, m, CH_2_) (protons of NH groups invisible); MS (ESI) *m/z*: 428.1, 239.2, 185.1; MS-HR (ESI) calcd for C_22_H_22_Cl_2_N_4_O: 428.11707, found: 428.11843.

#### 5,6-Dichloro-N-[5-(2,3-dihydro-1H-cyclopenta[b]quinolin-9-ylamino)-pentyl]-nicotinamide (2d)

Yield: 76%; beige solid; mp 80–83 °C; IR (KBr) ν (cm^−1^): 752.4; 1250.7; 1365.8; 1411.2; 1538.2; 2931.3; 3259.2; ^1^H NMR (600 MHz, CD_3_OD) δ 8.63 (1H, s, Ar), 8.28 (2H, d, *J* = 8.7 Hz, Ar) 8.24 (1H, s, Ar), 7.83 (1H, *t*, *J* = 8.2 Hz, Ar), 7.71 (1H, d, *J* = 8.8 Hz, Ar), 7.60 (1H, *t*, *J* = 8.4 Hz, Ar), 3.85 (2H, *t*, *J* = 7.1 Hz, CH_2_), 3.43 (4H, dt, *J* = 24.5, 7.1 Hz, CH_2_), 3.19 (2H, *t*, *J* = 7.9 Hz, CH_2_), 2.31 (2H, p, *J* = 7.8 Hz, CH_2_), 1.85 (2H, p, *J* = 15.1, 7.4 Hz, CH_2_), 1.74 (2H, p, *J* = 7.0 Hz, CH_2_), 1.56 (2H, p, *J* = 7.7, 7.2 Hz, CH_2_) (protons of NH groups invisible); MS (ESI) *m/z*: 442.1, 253.2, 185.1; MS-HR (ESI) calcd for C_23_H_24_Cl_2_N_4_O: 442.13272, found: 442.13243.

#### 5,6-Dichloro-N-[6-(2,3-dihydro-1H-cyclopenta[b]quinolin-9-ylamino)-hexyl]-nicotinamide (2e)

Yield: 62%; beige solid; mp 83–84 °C; IR (KBr) ν (cm^−1^): 753.3; 1229.6; 1361.9; 1411.1; 1539.7; 2932.2; 3234.4; ^1^H NMR (600 MHz, CD_3_OD) δ 8.70 (1H, s, Ar), 8.33 (1H, s, Ar), 8.26 (1H, d, *J* = 8.2 Hz, Ar), 7.79 (1H, *t*, *J* = 7.7 Hz, Ar), 7.74 (1H, d, *J* = 8.3 Hz, Ar), 7.57 (1H, *t*, *J* = 7.7 Hz, Ar), 3.79 (2H, *t*, *J* = 7.2 Hz, CH_2_), 3.42 (2H, *t*, *J* = 7.1 Hz, CH_2_), 3.37 (2H, *t*, *J* = 7.3 Hz, CH_2_), 3.14 (2H, *t*, *J* = 7.9 Hz, CH_2_), 2.27 (2H, p, *J* = 7.8 Hz, CH_2_), 1.79 (2H, p, *J* = 7.6, 7.2 Hz, CH_2_), 1.68 (2H, p, *J* = 7.3 Hz, CH_2_), 1.57–1.46 (4H, m, CH_2_) (protons of NH groups invisible); MS (ESI) *m/z*: 456.2, 267.2, 185.1; MS-HR (ESI) calcd for C_24_H_26_Cl_2_N_4_O: 456.14837, found: 456.15006.

#### 5,6-Dichloro-N-[7-(2,3-dihydro-1H-cyclopenta[b]quinolin-9-ylamino)-heptyl]-nicotinamide (2f)

Yield: 64%; beige solid; mp 70–74 °C; IR (KBr) ν (cm^−1^): 755.9; 1227.9; 1359.5; 1411.9; 1526.2; 2931.8; 3247.0; ^1^H NMR (600 MHz, CD_3_OD) δ 8.71 (1H, s, Ar), 8.33 (1H, s, Ar), 8.24 (1H, d, *J* = 3.4 Hz, Ar), 8.23 (1H, d, *J* = 3.4 Hz, Ar), 7.75 (1H, *t*, *J* = 8.6 Hz, Ar), 7.56 (1H, *t*, *J* = 7.3 Hz, Ar), 3.80–3.74 (2H, m, CH_2_), 3.43–3.37 (4H, m, CH_2_), 3.13 (2H, *t*, *J* = 7.8 Hz, CH_2_), 2.30–2.21 (2H, m, CH_2_), 1.77 (2H, m, CH_2_), 1.72–1.60 (2H, m, CH_2_), 1.56–1.45 (6H, m, CH_2_) (protons of NH groups invisible); MS (ESI) *m/z*: 470.2, 281.2, 185.1; MS-HR (ESI) calcd for C_25_H_30_Cl_2_N_4_O: 470.16402, found: 470.16412.

#### 5,6-Dichloro-N-[8-(2,3-dihydro-1H-cyclopenta[b]quinolin-9-ylamino)-octyl]-nicotinamide (2g)

Yield: 76%; beige solid; mp 74–75 °C; IR (KBr) ν (cm^−1^): 755.2; 1227.7; 1357.9; 1411.8; 1518.8; 2929.3; 3242.1; ^1^H NMR (600 MHz, CD_3_OD) δ 8.72 (1H, s, Ar), 8.35 (1H, s, Ar), 8.10 (1H, d, *J* = 8.7 Hz, Ar), 7.75 (1H, d, *J* = 8.4 Hz, Ar), 7.61 (1H, *t*, *J* = 7.7 Hz, Ar), 7.42 (1H, *t*, *J* = 7.7 Hz, Ar), 3.69–3.63 (2H, m, CH_2_), 3.40–3.35 (2H, m, CH_2_), 3.31–3.25 (2H, m, CH_2_), 3.02 (2H, *t*, *J* = 7.8 Hz, CH_2_), 2.18 (2H, p, *J* = 7.6 Hz, CH_2_), 1.70 (2H, p, *J* = 7.4 Hz, CH_2_), 1.60–1.65 (2H, m, CH_2_), 1.31–1.45 (8H, m Hz, CH_2_) (protons of NH groups invisible); MS (ESI) *m/z*: 484.2, 295.2, 185.1; MS-HR (ESI) calcd for C_26_H_30_Cl_2_N_4_O: 484.17967, found: 484.18029.

#### 5,6-Dichloro-N-[9-(2,3-dihydro-1H-cyclopenta[b]quinolin-9-ylamino)-nonyl]-nicotinamide (2h)

Yield: 68%; beige solid; mp 73–75 °C; IR (KBr) ν (cm^−1^): 754.6; 1211.6; 1358.7; 1425.1; 1522.1; 2928.6; 3204.8; ^1^H NMR (600 MHz, CD_3_OD) δ 8.72 (1H, s, Ar), 8.35 (1H, s, Ar), 8.19 (1H, d, *J* = 8.5 Hz, Ar), 7.75 (1H, d, *J* = 8.3 Hz, Ar), 7.71 (1H, *t*, *J* = 7.5 Hz, Ar), 7.51 (1H, *t*, *J* = 7.7 Hz, Ar), 3.72 (2H, *t*, *J* = 7.3 Hz, CH_2_), 3.38 (2H, *t*, *J* = 7.0 Hz, CH_2_), 3.31–3.35 (2H, m, CH_2_), 3.09 (2H, *t*, *J* = 7.8 Hz, CH_2_), 2.19–2.25 (2H, m, CH_2_), 1.73 (2H, p, *J* = 7.2 Hz, CH_2_), 1.60–1,64 (2H, m, CH_2_), 1.42–1,50 (10H, m, CH_2_) (protons of NH groups invisible); MS (ESI) *m/z*: 498.2, 309.2, 185.1; MS-HR (ESI) calcd for C_27_H_32_Cl_2_N_4_O: 498.19532, found: 498.19592.

### General procedure for the synthesis of compounds 3a–3h

Compounds **2a** (0.020 g, 0.050 mM), **2b** (0.020 g, 0.048 mM), **2c** (0.020 g, 0.047 mM), **2d** (0.020 g, 0.045 mM), **2e** (0.020 g, 0.044 mM), **2f** (0.020 g, 0.043 mM), **2g** (0.020 g, 0.041 mM), and **2h** (0.020 g, 0.040 mM), were dissolved in methanol (1 ml). Then, 4 ml of HCl/ether was added to the mixture and the reaction was stirred for 10 min. After 24 h, precipitates were formed, isolated by filtration and dried. In this synthesis **3a**–**3h** compounds were obtained. Physical and spectral data are listed below.

#### 5,6-Dichloro-N-[2-(2,3-dihydro-1H-cyclopenta[b]quinolin-9-ylamino)-ethyl]-nicotinamide hydrochloride (3a)

Yield: 43%; brown solid; mp 180–182 °C; IR (KBr) ν (cm^−1^): 759.1; 1228.3; 1371.2; 1414.7; 1544.5; 2931.1; 3219.5; ^1^H NMR (600 MHz, DMSO-d6) δ 14.02 (1H, s, HCl), 9.19 (1H, t, *J* = 5.7 Hz, NH), 8.77 (1H, s, Ar), 8.50 (1H, d, *J* = 8.5 Hz, Ar), 8.45 (1H, s, Ar), 7.88 (1H, d, *J* = 7.9 Hz, Ar), 7.83 (1H, d, *J* = 7.6 Hz, Ar), 7.66 (1H, d, *J* = 8.3 Hz, Ar), 4.08 (1H, s, NH), 3.95 (2H, q, *J* = 6.4 Hz, CH_2_), 3.61 (2H, q, *J* = 6.2 Hz, CH_2_), 3.31–3.36 (2H, m, CH_2_), 3.15 (2H, *t*, *J* = 7.9 Hz, CH_2_), 2.19 (2H, p, *J* = 7.7 Hz, CH_2_) (protons of NH groups invisible); MS (ESI) *m/z*: 400.1, 217.0, 185.1; MS-HR (ESI) calcd for C_20_H_19_Cl_3_N_4_O: 400.08577, found: 400.08590.

#### 5,6-Dichloro-N-[3-(2,3-dihydro-1H-cyclopenta[b]quinolin-9-ylamino)-propyl]-nicotinamide hydrochloride (3b)

Yield: 36%; beige solid; mp 206–207 °C; IR (KBr) ν (cm^−1^): 754.4; 1226.5; 1358.5; 1420.7; 1540.1; 2944.9; 3217.5; ^1^H NMR (600 MHz, DMSO-d6) δ 13.86 (1H, s, HCl), 8.96 (1H, *t*, *J* = 5.6 Hz, NH), 8.77 (1H, s, Ar), 8.44–8.47 (2H, m, Ar), 7.87 (1H, *t*, *J* = 8.0 Hz, Ar), 7.78 (1H, d, *J* = 8.3 Hz, Ar), 7.63 (1H, *t*, *J* = 7.7 Hz, Ar), 4.08 (1H, s, NH), 3.83 (2H, q, *J* = 6.7 Hz, CH_2_), 3.44 (2H, q, *J* = 6.3 Hz, CH_2_), 3.26–3.35 (2H, m, CH_2_), 3.14 (2H, *t*, *J* = 7.9 Hz, CH_2_), 2.15 (2H, p, *J* = 7.7 Hz, CH_2_), 1.98 (4H, p, *J* = 7.0 Hz, CH_2_) (protons of NH groups invisible); MS (ESI) *m/z*: 414.1, 231.0, 185.1; MS-HR (ESI) calcd for C_21_H_21_Cl_3_N_4_O: 400.08577, found: 400.08590.

#### 5,6-Dichloro-N-[4-(2,3-dihydro-1H-cyclopenta[b]quinolin-9-ylamino)-butyl]-nicotinamide hydrochloride (3c)

Yield: 56%; beige solid; mp 139–141 °C; IR (KBr) ν (cm^−1^): 754.7; 1227.3; 1362.3; 1415.1; 1538.2; 2937.4; 3234.7; ^1^H NMR (600 MHz, DMSO-d6) δ 13.92 (1H, s, HCl), 8.85 (1H, *t*, *J* = 5.5 Hz, NH), 8.77 (1H, s, Ar), 8.48 (1H, d, *J* = 8.5 Hz, Ar), 8.46 (1H, s, Ar), 7.87 (1H, *t*, *J* = 7.6 Hz, Ar), 7.80 (1H, d, *J* = 8.3 Hz, Ar), 7.64 (1H, *t*, *J* = 7.7 Hz, Ar), 4.02 (1H, s, NH), 3.76 (2H, q, *J* = 6.7 Hz, CH_2_), 3.38–3.33 (4H, m, CH_2_), 3.14 (2H, *t*, *J* = 7.9 Hz, CH_2_), 2.16 (2H, p, *J* = 7.7 Hz, CH_2_), 1.75 (2H, p, *J* = 6.8 Hz, CH_2_), 1.67 (2H, p, *J* = 6.8 Hz, CH_2_) (protons of NH groups invisible); MS (ESI) *m/z*: 428.1, 239.2, 185.1; MS-HR (ESI) calcd for C_22_H_23_Cl_3_N_4_O: 428.11707, found: 428.11698.

#### 5,6-Dichloro-N-[5-(2,3-dihydro-1H-cyclopenta[b]quinolin-9-ylamino)-pentyl]-nicotinamide hydrochloride (3d)

Yield: 50%; brown solid; mp 120–122 °C; IR (KBr) ν (cm^−1^): 756.7; 1225.6; 1354.4; 1420.4; 1563.6; 2936.1; 3218.6; ^1^H NMR (600 MHz, DMSO-d6) δ 13.81 (1H, s, HCl), 8.73–8.76 (2H, m, Ar, NH), 8.44 (1H, d, *J* = 8.5 Hz, Ar), 8.41 (1H, s, Ar), 7.86 (1H, *t*, *J* = 7.6 Hz, Ar), 7.77 (1H, d, *J* = 8.1 Hz, Ar), 7.62 (1H, *t*, *J* = 8.0 Hz, Ar), 4.08 (1H, s, NH), 3.73 (2H, q, *J* = 6.7 Hz, CH_2_), 3.29–3.33 (4H, m, CH_2_), 3.14 (2H, *t*, *J* = 7.9 Hz, CH_2_), 2.18 (2H, p, *J* = 7.8 Hz, CH_2_), 1.72 (2H, p, *J* = 7.6 Hz, CH_2_), 1.61 (2H, p, *J* = 6.9 Hz, CH_2_), 1.44 (2H, p, *J* = 8.4 Hz, CH_2_) (protons of NH groups invisible); MS (ESI) *m/z*: 442.1, 253.2, 185.1; MS-HR (ESI) calcd for C_23_H_25_Cl_3_N_4_O: 442.13272, found: 442.13368.

#### 5,6-Dichloro-N-[6-(2,3-dihydro-1H-cyclopenta[b]quinolin-9-ylamino)-hexyl]-nicotinamide hydrochloride (3e)

Yield: 53%; beige solid; mp 97–99 °C; IR (KBr) ν (cm^−1^): 755.6; 1229.4; 1359.5; 1426.1; 1584.6; 2936.2; 3234.9; ^1^H NMR (600 MHz, DMSO-d6) δ 13.79 (1H, s, HCl), 8.79 – 8.73 (2H, m, Ar, NH), 8.48–8.43 (1H, m, Ar), 7.88 (1H, *t*, *J* = 7.8 Hz, Ar), 7.79 (1H, d, *J* = 7.9 Hz, Ar), 7.65 (1H, *t*, *J* = 8.2 Hz, Ar), 4.07 (1H, s, NH), 3.72 (2H, q, *J* = 7.0 Hz, CH_2_), 3.24–3.33 (4H, m, CH_2_), 3.14 (2H, *t*, *J* = 7.8 Hz, CH_2_), 2.18 (2H, p, *J* = 7.8, 7.1 Hz, CH_2_), 1.69 (2H, p, *J* = 15.2, 7.4 Hz, CH_2_), 1.56 (2H, p, *J* = 15.5, 7.5 Hz, CH_2_), 1.45–1.36 (2H, m, CH_2_) (protons of NH groups invisible); MS (ESI) *m/z*: 456.1, 267.2, 185.1; MS-HR (ESI) calcd for C_24_H_27_Cl_3_N_4_O: 456.14837, found: 456.14885.

#### 5,6-Dichloro-N-[7-(2,3-dihydro-1H-cyclopenta[b]quinolin-9-ylamino)-heptyl]-nicotinamide hydrochloride (3f)

Yield: 57%; beige solid; mp 100–102 °C; IR (KBr) ν (cm^−1^): 757.5; 1228.1; 1360.1; 1424.3; 1557.4; 2929.8; 3227.7; ^1^H NMR (600 MHz, DMSO-d6) δ 13.82 (1H, s, HCl), 8.80–8.75 (2H, m, Ar, NH), 8.48–8.43 (1H, m, Ar), 7.88 (1H, *t*, *J* = 7.4 Hz, Ar), 7.80 (1H, d, *J* = 8.1 Hz, Ar), 7.65 (1H, *t*, *J* = 7.6 Hz, Ar), 5.33 (1H, s, NH), 3.72 (2H, q, *J* = 6.9 Hz, CH_2_), 3.26–3.28 (4H, m, CH_2_), 3.15 (2H, *t*, *J* = 7.9 Hz, CH_2_), 2.18 (2H, p, *J* = 7.7 Hz, CH_2_), 1.72–1.64 (2H, m, CH_2_), 1.52–1.56 (2H, p, *J* = 13.5, 6.1 Hz, CH_2_), 1.34–1.41 (4H, m, CH_2_) (protons of NH groups invisible); MS (ESI) *m/z*: 470.1, 281.1, 185.1; MS-HR (ESI) calcd for C_25_H_29_Cl_3_N_4_O: 470.16402, found: 470.16425.

#### 5,6-Dichloro-N-[8-(2,3-dihydro-1H-cyclopenta[b]quinolin-9-ylamino)-octyl]-nicotinamide hydrochloride (3g)

Yield: 56%; beige solid; mp 97–98 °C; IR (KBr) ν (cm^−1^): 761.9; 1227.8; 1358.7; 1422.6; 1557.7; 2926.3; 3220.6; ^1^H NMR (600 MHz, DMSO-d6) δ 13.84 (1H, s, HCl), 8.80–8.74 (2H, m, Ar, NH), 8.49–8.44 (1H, m, Ar), 7.88 (1H, *J* = 7.7 Hz, Ar), 7.80 (1H, d, *J* = 7.9 Hz, Ar), 7.65 (1H, *t*, *J* = 7.7 Hz, Ar), 5.34 (1H, s, NH), 3.71 (2H, q, *J* = 6.7 Hz, CH_2_), 3.25–3.29 (4H, m, CH_2_), 3.15 (2H, *t*, *J* = 7.9 Hz, CH_2_), 2.18 (2H, p, *J* = 7.7 Hz, CH_2_), 1.68 (2H, p, *J* = 8.1, 7.6 Hz, CH_2_), 1.53 (2H, p, *J* = 6.9, 5.7 Hz, CH_2_), 1.36–1.41 (6H, m, CH_2_) (protons of NH groups invisible); MS (ESI) *m/z*: 484.2, 295.2, 185.1; MS-HR (ESI) calcd for C_26_H_31_Cl_3_N_4_O: 484.17967, found: 484.1805.

#### 5,6-Dichloro-N-[9-(2,3-dihydro-1H-cyclopenta[b]quinolin-9-ylamino)-nonyl]-nicotinamide hydrochloride (3h)

Yield: 37%; beige solid; mp 88–89 °C; IR (KBr) ν (cm^−1^): 757.9; 1227.9; 1359.7; 1424.3; 1557.9; 2925.6; 3223.4; ^1^H NMR (600 MHz, DMSO-d6) δ 13.97 (1H, s, HCl), 8.78–8.81 (2H, m, Ar, NH), 8.51–8.46 (1H, m, Ar), 7.86–7.89 (1H, m, Ar), 7.83 (1H, d, *J* = 8.2 Hz, Ar), 7.65 (1H, *t*, *J* = 7.7 Hz, Ar), 3.92 (1H, s, NH), 3.71 (2H, q, *J* = 6.8 Hz, CH_2_), 3.25–3.29 (4H, m, CH_2_), 3.15 (2H, *t*, *J* = 7.9 Hz, CH_2_), 2.18 (2H, p, *J* = 7.7 Hz, CH_2_), 1.67 (2H, p, *J* = 7.8 Hz, CH_2_), 1.52 (2H, p, *J* = 6.7 Hz, CH_2_), 1.35–1.41 (8H, m, CH_2_) (protons of NH groups invisible); MS (ESI) *m/z*: 498.2, 309.2, 185.1; MS-HR (ESI) calcd for C_27_H_33_Cl_3_N_4_O: 498.19532, found: 498.18591.

### In vitro inhibition studies on AChE and BuChE

The inhibitory activities of the tested compounds towards AChE and BuChE were performed using Ellman’s method^25^. All reagents including AChE (EC 3.1.1.7, from *Electrophorus electricus*), BuChE (EC 3.1.1.8, from equin serum), DTNB (Ellman’s reagent), and acetylthiocholine iodide (ATC iodide) were purchased from Sigma Aldrich (Steinheim, Germany). Analysis was executed in following procedure: AChE and BuChE solution was made by dissolving enzymes in phosphate buffer (pH 7.4) to obtain 2 and 4 U/ml, respectively. Stock solutions of the tested compounds were prepared in phosphate buffer (pH 7.4) with nine concentrations in the range of 0.009–2.3 µM. The study was performed in triplicate and recorded in 96-well microplates, using multifunctional microplate reader. The assay medium contained DTNB solution 0.4 mg/ml (76 µl), AChE (10 µl), various concentration of the examined compounds (14 µl) and ATC iodine solution (40 µl) at the concentration of 1 mM. For the reference value, 14 µl of the test compound solutions were replaced by phosphate buffer (pH 7.4). Next microplates were incubated at room temperature and the absorption (412 nm) was measured after 10 min for the plates with AChE and after 8 min for BuChE. Inhibition activity was expressed as a percentage of the activity of the tested compound relative to the reference control. The obtained IC_50_ values expressed 50% inhibition activity against AChE or BuChE.

### Kinetic characterisation of AChE inhibition

The kinetic characterisation was evaluated according to the Ellman’s method^4^ ^,^ ^25^. Plots of 1/velocity versus reciprocal substrate concentration were constructed at different final concentrations of the ATC iodide in the range of 100–10 µM gradually decreased by 10 µM. The studies were performed using two final concentrations of **3b** inhibitor (1.0, 0.1 µM) and without this inhibitor. The tested medium contained: DTNB solution (0.4 mg/ml), AChE solution (2 U/ml), solution of the examined compound and different concentrations of the substrate ACT iodide in a total volume of 140 µM. Absorbance were detected at 412 nm at room temperature after 6 min. Received date were calculated and presented on the Lineweaver–Burk plot. Determinated the K_M_ and V_max_ values were used to define the type of enzyme inhibition.

### Kinetic characterisation of BuChE inhibition

Kinetic analysis was performed using Ellman’s test^4^ ^,^ ^25^. Plots of reciprocal velocity versus reciprocal substrate concentration were made at various final concentrations of the ATC iodide in the range of 100–10 µM gradually decreased by 10 µM. The analysis were prepared for three final concentrations of **3b** inhibitor (1.11, 0.22, 0.56 µM) and without inhibitor. Total volume of tested medium (140 µl) comprised: DTNB solution (0.4 mg/ml), BuChE solution (4 U/ml), solution of the examined compound and different concentrations of the substrate ATC iodide. Absorbance was detected at 412 nm at room temperature after 8 min. Results were calculated and presented on the Lineweaver–Burk plot. Received K_M_ and V_max_ values allowed to define the type of enzyme inhibition.

### Beta-amyloid assay

Recent finding about decline in AD suggest a relationship between amyloid β peptide (Aβ) accumulation and the neurodegenerative process. The Aβ_1–42_ aggregation assay was established using the Thioflavin T fluorescence method^22^ ^,^ ^26^ ^,^ ^27^. Experiment was performed by incubating the peptides previously dissolved in DMSO at room temperature for 24 h (final Aβ_1–42_ concentration – 12.58 μM) with or without the tested compounds at the different concentrations (5, 10, 25, 50, and100 μM). After incubation all samples were diluted in PBS – phosphate buffer (pH 7.4) containing Thioflavin T (5 μM). Subsequently, after 5 min incubation with the dye, fluorescence signal was measured (excitation wavelength 446 nm, emission wavelength 490 nm) on a monochromator based multimode microplate reader (Synergy H1, Bio-Tek, Winooski, VT), adapted for 96-well microtiter plates. Next the percent of inhibition on aggregation was calculated by the following expression: 100 – (IF_i_/IF_o_ × 100). IF_i_ and IF_o_ were the fluorescence intensities obtained for absorbance in the presence and absence of inhibitors, respectively. The background values were deducted in the calculations.

### Cell culture and cytotoxicity assay

In the experiment, ATCC CCL-185 cell line was used (Epithelial cells from human lungs, carcinoma). Cells were seeded on 96-wells plate (F12 Thermo Fisher medium, Waltham MA) and incubated for 24 h. Then the cells were treated with various concentrations (0.22, 1.11, 2.22, 22.22, and 222.20 µM) of the tested compound dissolved in methanol and incubated for 48 h. Final methanol concentration in each well was 1 µl per 100 µl of medium. As a control, the same medium as for cells breeding was used with addition of 1 µl methanol and without studied compound. As a positive control pure methanol was used. After the time of incubation, procedure was carried out according to Vybrant MTT Cell Proliferation Assay Kit: cells were labelled with 10 µl of the 12 mM MTT stock solution, incubated at 37 °C for 4 h, all medium but 25 µl was removed from the wells, 50 µl of DMSO was added, incubated for 10 min at 37 °C and the absorbance at 540 nm was measured^28–30^.

Assay was performed on human liver cells which were purchased from the American Type Culture Collection (Rockville, MD). The cells were maintained in Dulbecco’s modified Eagle’s medium (DMEM) supplemented with 5% foetal bovine serum, 50 U/ml penicillin, and 50 μg/ml streptomycin at 37 °C in a 5% CO_2_ incubator. Cells were incubated for 24 h in DMEM with varying concentrations of tacrine and **3b** compound. Toxicity of tested substances was determined by the method of Carmichael et al.^31^. Liver cells were maintained as described above in 24-well plates. Cells were incubated for 24 h with varying concentrations of tacrine and **3b** compound, after that medium was discarded, and the cells were rinsed three times with phosphate-buffered saline (PBS). Then, the cells were incubated for 30 min in 1 ml of PBS with 25 μl of MTT (5 mg/ml). Medium was removed from the wells, and the cells were lysed 1 ml of DMSO with 20 μl of Sorensen’s buffer (0.1 M glycine with 0.1 M NaCl, pH 10.5). The absorbance was measured at the wavelength of 570 nm. Component-treated cells were calculated as a percent of each control cell lines.

### Hyaluronidase inhibition test

The inhibition study of hyaluronidase was carried out by turbidimetric method adapted to the 96-well plates and described previously by Michel et al^32^ ^,^ ^33^. At the beginning, 20 µl of the tested **3b** compound solution in monosodium phosphate buffer (pH 7.0) with 40 µl of hyaluronidase solution (22.55 U/ml, hyaluronidase from bovine testes Type I-S, Sigma Aldrich, Steinheim, Germany) were added to the wells of 96-well microtiter plates. Mixture was incubated in the dark at 37 °C for 10 min. Then, 40 µl of hyaluronic acid solution (0.03%, Sigma Aldrich, Steinheim, Germany) in monosodium phosphate buffer (pH 5.35) was added to the wells and the plate was incubated in the dark at 37 °C for 45 min. At the end, 300 µl of bovine serum albumin solution (0.1%, Serva) in sodium acetate buffer (pH 3.75) was added to the wells and incubated in the room temperature for 10 min. The changes in turbidity were measured at 600 nm by a microplate reader (BioTek, Winooski, VT). The assay was run out in three experiments in triplicate to calculate IC_50_ values. Heparin (WZF, Polfa) was used as a positive control^34^.

The inhibition of hyaluronidase by the tested compound was calculated following the equation:

$$\%\;inhibition=100x\left(1-\left(\frac{A_{HA}-A_{AN}}{A_{HA}-A_{HYAL}}\right)\right)$$

where A_HA_ − absorbance of solution without the enzyme (positive control),

A_HYAL_ − absorbance of solution without the tested compound (negative control), A_AN_ − absorbance of solution with the tested compound.

### Molecular modelling

Acetyl- and butyrylcholinesterase structures were retrieved from Protein Data Bank (PDB codes: 2CKM and 1P0I, respectively). Before docking each protein was prepared in the following way: protonation of all histidine residues were set to Nε, all hydrogen atoms were added, ligand and water molecules were removed, and all amino acid residues within radius of 10 Å from bis-(7)-tacrine for AChE and 20 Å from the glycerol molecule present in the active centre of BChE were defined as the binding site. Docking was performed with GoldSuite 5.1 (CCDC)^35^ using standard settings of the genetic algorithm with population size 100, 5 islands, and 100,000 operations^4^ ^,^ ^36^. Three-dimensional structures of all ligands were created with Corina on-line (Molecular Networks GmbH, Nürnberg, Germany)^37^ was used to check atom types and assign Gasteiger–Marsili charges^38^. Ten poses for each ligand were collected and sorted by GoldScore (for AChE) and ChemScore (for BChE) function value. PyMOL 0.99rc6 (DeLano Scientific LLC, Philadelphia, PA)^39^ was used to visualise the results of docking.

### ADMET analysis

The drug discovery is a time consuming and complex process requiring multi-disciplinary approaches to develop riskless and effective medicines. ADMET (Absorption, Distribution, Metabolism, Excretion, and Toxicity) predictions are important initial steps towards the development of novel pharmaceuticals in the fight against Alzheimer’s disease. However, it has been reported that 95% of drug candidate molecules fail in the development stages, and 50% of such failures are caused by unsatisfactory ADMET properties. To avoid this failure, we calculated among others for all the compounds, as well as tacrine, the parameters that define the “Rule of Five” which the drugs in general follow: molecular weight lower than 500, number of hydrogen bond donors lower or equal 5, number of hydrogen bond acceptors lower or equal than 10 and log *p* lower than 5. It is necessary to develop *in silico* methods that are faster, simpler and more cost-effective for evaluating the ADMET properties of a molecule in advance. The ADMET filtering was done with the help of ACD/Percepta version 14.0.0 (Advanced Chemistry Development, Inc., Metropolitan Toronto, Canada)^40–42^.

## Results and discussion

### Chemistry

**Scheme 1.**
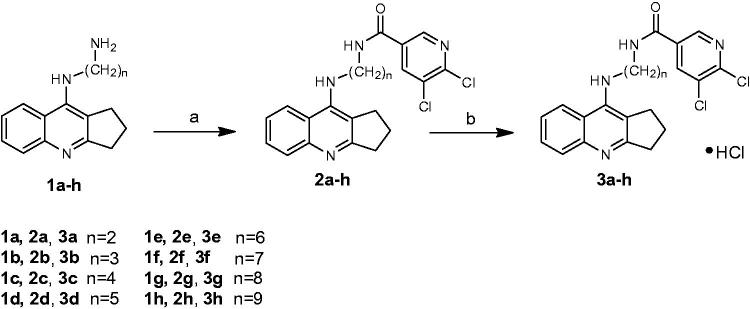
Synthesis of compounds **2a**–**2h** and **3a**–**3h**. Reagents: (a) 5,6-dichloronicotynic acid, CDMT, N-methylmorpholine, THF; (b) HCl/ether.

The synthesis of the final compounds was accomplished as illustrated in Scheme 1. Novel, multifunctional derivatives consists of 2,3-dihydro-1H-cyclopenta[b]quinoline with 5,6-dichloronicotinic acid moiety using diamine linker have been carried out in two steps. In order to obtain end products we have used intermediates **1a**–**1h** based on reactions developed and published earlier^16^. New compounds were obtained via the synthesis between 5,6-dichloronicotinic acid, previously activated by 2-chloro-4,6-dimethoxy-1,3,5-triazine (CDMT), dropwise addition of N-methylmorpholine and reacted with compounds **1a**–**1h** dissolved in tetrahydrofuran at −5 °C. Monitoring the reactions using TLC showed the reactions were completed after 2h. Compounds **2a**–**2h** were obtained with satisfactory yield (62–85%, mean 74%) and purified by flash chromatography. The last step of the synthesis involved conversion of the obtained compounds **2a**–**2h** into hydrochlorides **3a**–**3h**. Compounds **2a**–**2h** were dissolved in a small volume of methanol and next HCl in ether was added.

### Biological activity

To estimate the inhibitory activity towards AChE (from electric eel) and BuChE (from equine serum) of new derivatives **3a**–**3h**, Ellman’s test was performed. As a reference compounds were used tacrine and donepezil. The IC_50_ values of *Ee*AChe and *Eq*BuChE inhibition is collected in Table 1. All synthesised compounds are active AChE inhibitors with IC_50_ values in the range of 0.052–0.744 µM. In comparison with tacrine only two compounds (**3c**, **3d**) were less active. Taking donepezil (0.103 µM) into consideration, **3a** compound (0.065 µM), **3b** (0.052 µM), and **3e** (0.053 µM) showed higher potency. Analysis showed no significant relationship between structure and the obtained IC_50_ values. Among the described compounds, **3b** (0.052 µM) revealed the highest inhibitory potency against AChE, which in comparison to tacrine and donepezil, was about 3-times and 2-times higher, respectively. Consequently, compound **3b** was chosen for the kinetic analysis of the AChE and BuChE inhibition.

**Table 1.** The activity of novel compounds **3a–3h** against acetylcholinesterase from electric eel and equine butyrylcholinesterase.

Compound	AChE IC_50_ ± SEM (µM)^a^	BuChE IC_50_ ± SEM (µM)^b^	Selectivity for AChE^c^	Selectivity for BuChE^d^
**3a**	0.065 ± 0.007	1.863 ± 0.083	28.593	0.035
**3b**	0.052 ± 0.002	0.158 ± 0.029	3.030	0.330
**3c**	0.744 ± 0.046	0.797 ± 0.086	1.071	0.934
**3d**	0.285 ± 0.038	0.460 ± 0.038	1.615	0.619
**3e**	0.053 ± 0.005	0.127 ± 0.014	2.380	0.420
**3f**	0.125 ± 0.021	0.071 ± 0.012	0.569	1.756
**3g**	0.152 ± 0.045	0.108 ± 0.006	0.711	1.407
**3h**	0.155 ± 0.045	0.082 ± 0.030	0.529	1.889
Donepezil	0.103 ± 0.016	11.826 ± 2.060	114.971	0.009
Tacrine	0.163 ± 0.041	0.020 ± 0.003	0.122	8.187

a IC_50_: 50% inhibitory concentration (means ± SEM of three independent experiments) of AChE.

b IC_50_: 50% inhibitory concentration (means ± SEM of three independent experiments) of BuChE.

c Selectivity for AChE: IC_50_(BuChE)/IC_50_(AChE).

d Selectivity for BuChE: IC_50_(AChE)/IC_50_(BuChE).

All derivatives were also very active BuChE inhibitors and their IC_50_ values ranged from 0.071 to 1.863 µM. The whole series revealed significant higher activity than donepezil (11.826 µM), although comparing with tacrine none of them showed higher activity. The most active compound (**3f**) towards BuChE with IC_50_ = 0.071 µM presented 167-fold higher than donepezil.

**Figure 1.**
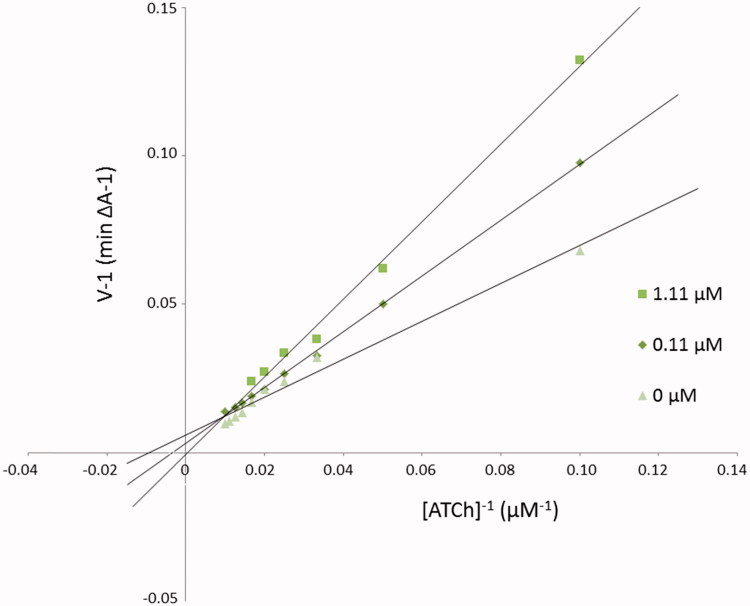
Lineweaver–Burk reciprocal plots illustrating mixed-type of *Ee*AChE inhibition by **3b** compound. ATCh: acetylthiocholine; V: initial velocity rate.

The mechanism of *Ee*AChE inhibition was investigated for inhibitor **3b** as the most valuable compound (IC_50_ = 0.052 µM) (Table 1). Lineweaver–Burk plot (Figure 1) presents 1/velocity versus 1/substrate concentrations in the range of 100–10 µM gradually decreased by 10 µM for different inhibitor **3b** concentrations (1.11, 0.11 µM) and without inhibitor. Based on Lineweaver–Burk plot’s analysis the mixed type of inhibition was elucidated. Lines crossing in the same point of coordinate system. At increasing inhibitor concentration K_M_ values (110.18, 293.78, and 2185.33 µM) and V_max_ values (166.67, 312.50, and 1666.67 A/min) also increased (Table 2).

**Table 2.** The V_max_ and K_M_ values at different **3b** inhibitor concentrations for AChE.

Concentration of inhibitor **3b** (µM)	K_M_ (µM)	Vmax (A/min)
1.11	2185.33	1666.67
0.11	293.78	312.50
0	110.18	166.67

**Figure 2.**
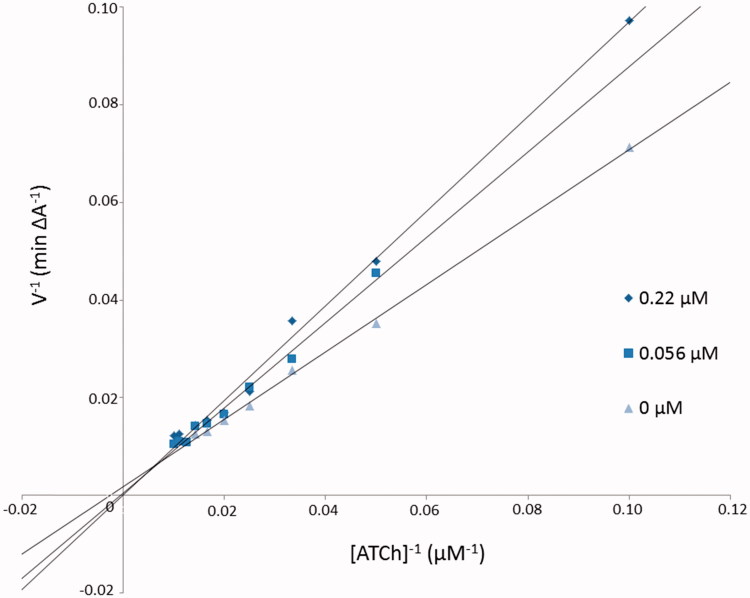
Lineweaver–Burk reciprocal plots illustrating mixed-type of *Eq*BuChE inhibition by **3b** compound. ATCh: acetylthiocholine; V: initial velocity rate.

The mechanism of *Eq*BuChE inhibition for inhibitor **3b** as the most potent compound was revealed from Lineweaver–Burk plot analysis (Figure 2). The reciprocal velocity was constructed as a function of reciprocal substrate concentration in the range 10–100 µM gradually decreased by 10 µM for different inhibitor **3b** concentrations (0.22, 0.56 µM) and without inhibitor. The analysis showed increased K_M_ values (10371.5, 3223.0, 1453.0, 536.5 µM) and increased Vmax values (3333.3, 1666.7, 769.2 A/min) with higher inhibitor concentrations (Table 3). Lines crossing in the same point in the Lineweaver–Burk plot revealed the mixed type of inhibition.

**Table 3.** The V_max_ and K_M_ values at different **3b** inhibitor concentrations for BuChE.

Concentration of inhibitor **3b** (µM)	K_M_ (µM)	Vmax (A/min)
0.22	3223.0	3333.3
0.56	1453.0	1666.7
0	536.5	769.2

**Figure 3.**
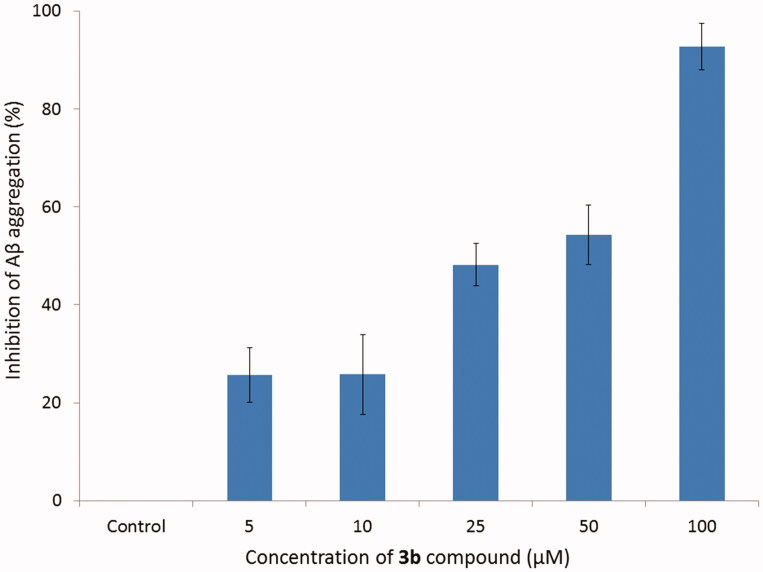
Inhibition of Aβ aggregation by compound **3b** at different concentrations. Thioflavin T assay (*λ*_exc_ = 446 nm; *λ*_em_ = 490 nm).

The results (summarised in Table 4) (Figure 3) show that the tested compound (**3b**) induced a decrease of the ThT fluorescence associated with the Aβ fibril binding. In all of presented concentrations **3b** compound presented aggregation inhibition values above 25%, whereas using 100 µM of inhibitor concentration presented the best activity (92.78%). At a concentration of 5 µM and 10 µM the tested compound showed the inhibition rate 25.67 and 25.76%, respectively. It means there is no significant difference; however, the effect is important. As the concentration increased, the inhibition rate also enhanced. At a concentration of 25 and 50 µM the inhibition rate reached the values of 48.18 and 54.30%, respectively. This relationship indicates dosage-dependent manner of the inhibition.

**Table 4.** Inhibition of Aβ aggregation at different concentrations of compound **3b**.

**3b** compound concentration (µM)	Aβ aggregation (%)	Inhibition of Aβ aggregation (%)
Control sample	100.00	0.00
5	74.31	25.69
10	74.23	25.77
25	51.82	48.18
50	45.70	54.30
100	7.22	92.78

**Figure 4.**
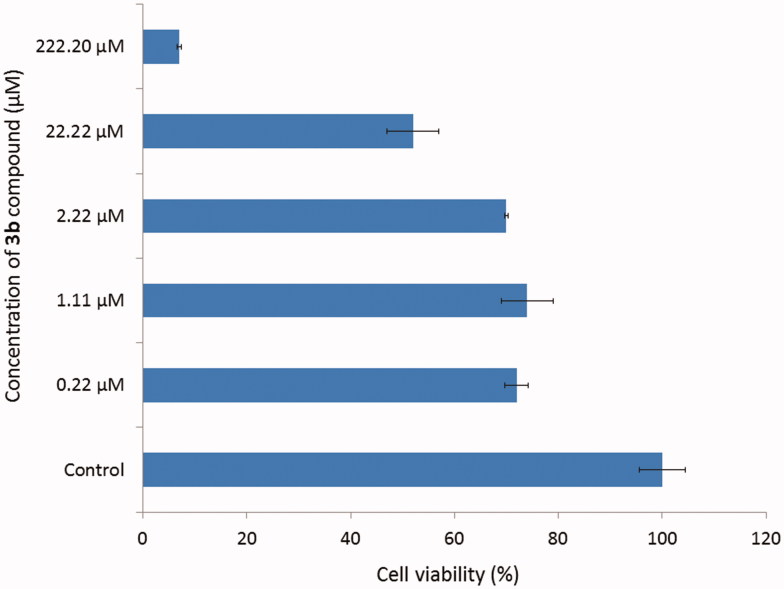
Effect of compound **3b** on the viability of cells.

Compound **3b** which showed the highest inhibitory activity towards AChE was further tested for the potential cytotoxic effect using ATCC CCL-185 cell line (Epithelial cells from human lungs, carcinoma). The cells are treated with novel compound at different concentration ranging from 0.22 to 222.20 µM and showed in Figure 4. The results were expressed as a percentage of cell viability, assuming control as 100%. The results revealed no significant different of cell viability (about 70%) in concentrations from 0.22 to 2.22 µM. Using 22.22 µM concentration of **3b** compound the cell viability suddenly decreased and in 222.20 µM concentration it reached only about 7%. To sum up, the concentration of **3b** derivative for 50% inactivation of AChE (0.052 µM) and BuChE (0.158 µM) is lower than cytotoxicity effect for this compound. Moreover, cytotoxicity assay performed on human liver cells showed no toxic effect of tested compounds.

Inflammation is a complex biological response to aggressive agents (pathogens, injury), involving local vascular system and immune system. The inflammation is regulated by anti-inflammatory mediators, including cytokines, chemokines, and by several cellular enzymes, such as hyaluronidase. Hyaluronidase is an enzyme responsible for hyaluronan depolymerisation and by this process; it weakens the integrity of tissues during the inflammation. The prolonged inflammatory process is commonly associated with the development of some chronic disease, such as AD or cancers. Mostly for the treatment of inflammatory diseases, the non-steroidal anti-inflammatory (NSAIDs) are in common use. Due to the many side effects, such as gastrointestinal, renal, and cardiovascular toxicity, their use should be limited^43^. Therefore, novel drugs with anti-inflammatory properties are being investigated.

**Figure 5.**
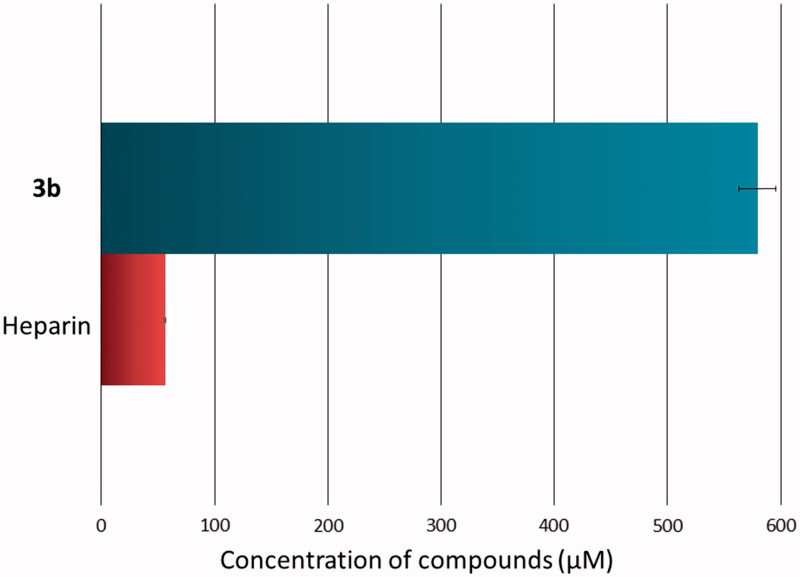
50% inhibition of hyaluronidase activity by **3b** compound and heparin.

The spectrophotometric assay was performed in order to determine the inhibitory effect of novel compound towards hyaluronidase^34^. The inhibitory effect was tested *in vitro* as a dose-dependent response. Table 5 presents IC_50_ values of novel compound and reference drug (hyaluronidase inhibitor) – heparin. **3b** compound presented high inhibitory activity towards hyaluronidase (IC_50_ 579.77 ± 16.28 µM), although heparin was stronger inhibitor (IC_50_ 56.41 ± 0.78 µM) (Figure 5). It can be conclude that **3b** compound has good anti-hyaluronidase activity and may decrease the risk of development of chronic disease.

**Table 5.** All values are presented as the means ± standard deviation (SD); IC_50_, 50% inhibition of enzyme activity.

Compound	HYAL IC_50_ ± SD (µM)
**3b**	579.77 ± 16.28
Heparin	56.41 ± 0.78

### Molecular modelling

**Figure 6.**
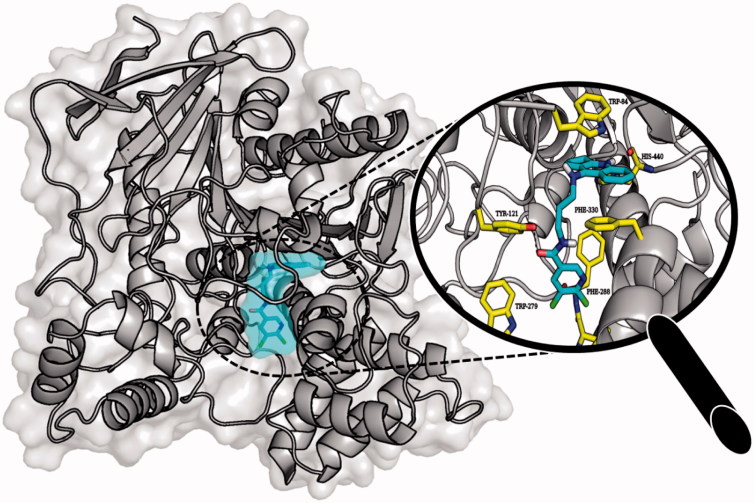
Binding mode of compound **3b** within the active site of AChE.

**Figure 7.**
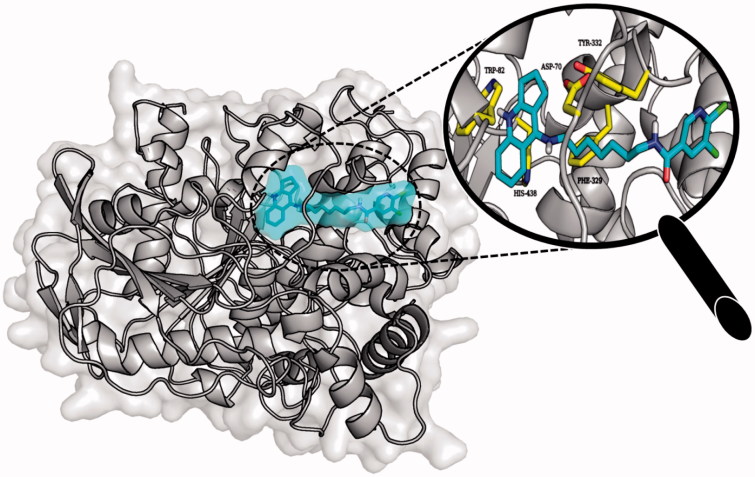
Binding mode of compound **3f** within the active site of BuChE.

Molecular modelling studies were performed in order to analyse the binding mode of the obtained compounds. In case of AChE tested ligands presented two conformations which were dependent on the length of the linker. Extended conformation was similar to the conformation of bis-(7)-tacrine bound to the enzyme while bent conformation corresponded to the conformation in which the phenyl ring was located near the wall of the entrance to the enzyme gorge. Ligands with short linkers occurred mostly in the bent arrangements. On the other hand longer linkers led to the extended orientation of the compounds. The most active derivative **3b** (Figure 6) is an example of the ligand with bent conformation. The cyclopentaquinoline moiety created a characteristic π–π stacking and cation-π interactions with Trp84 and Phe330. Tricyclic fragment was additionally stabilised by a hydrogen bond between the protonated nitrogen atom and oxygen atom from carbonyl group of His440 main chain. The linker was directed towards the gorge wall near the entrance where the hydroxyl group of Tyr121 participated in hydrogen bonding with ligand carbonyl oxygen. Chlorine-substituted pyridine ring was involved in π–π stacking interactions with Phe288. Docking studies confirmed that cyclopentaquinoline moiety mimicked the arrangement of former drug, tacrine in the active site. Tacrine received GoldScore value equal to 58.44. The most active compound **3b**, in the described conformation, was assessed by the same scoring function at level of 81.28 which stayed in accordance with results of biological studies. In the active site of BuChE similar conformations of ligands were observed. Compounds with shorter linker presented bent conformation located deep inside active site of enzyme. Molecules with longer linkers (6 and more carbon atoms) appeared in extended conformation providing contact with a peripheral anionic site. The binding mode of the most potent BuChE inhibitor **3f** is shown in Figure 7. Cyclopentaquinoline moiety participated in π–π stacking and cation-π interactions with Trp82. Long, aliphatic linker created hydrophobic interactions with Tyr332. Chlorine-substituted nicotinamide was located at the entrance to the binding site of the enzyme. That conformation was preferable for the most active BuChE inhibitors among the tested compounds. Comparing the most active inhibitor **3f** and tacrine, both cyclopentaquinoline and tetrahydroacridine moieties occupied the same area within the active site of BuChE and interacted in a similar way. The ChemScore value was equal to 106.78 for tacrine and 94.28 for compound **3f**, respectively, and corresponded to results of Ellman’s assay (see Table 1).

### ADMET analysis

Various pharmacokinetic and pharmacodynamics properties of compounds (absorption, distribution, metabolism, excretion, and toxicity) were studied using *in silico* ADMET profiling (Table 6). Physico-chemical indicators are increasingly used during the early stages of drug discovery to provide a comprehensive understanding of the key properties that affect biological functions (ADMET). Screening on the basis of blood brain barrier property is important for any lead molecule to act as potent inhibitor against Alzheimer’s disease. In particular, the new molecules should present a good CNS penetration profile and low toxic effects. The probability of positive Ames test for all new derivatives is much lower in comparison to the reference (tacrine) which means lower genotoxicity effect. Some of our compounds violated the “rule of five”, for **3e**, **3f**, **3g,** and **3h** compounds log *p* is higher than 5. In our study, all the tested compounds present good value of TPSA (≤90) so it is predicted to penetrate CNS. Furthermore, compounds presents sufficient brain penetration profile, log BB is not lower than −1. The results for our compounds are estimated at −0.12–0.55 which confirms previous results. . All the structures reported herein show suitable values (MW <460). To sum up, despite that compounds show good brain penetration profiles, it can be concluded that structure **3b** presents the best drug-like characteristics and ADMET properties of the series^44–46^.

**Table 6.** Absorption, distribution, metabolism, excretion, and toxicity (ADMET) parameters for tested compound and tacrine with the help of ACD/Percepta version 14.0.0.

	**3a**	**3b**	**3c**	**3d**	**3e**	**3f**	**3g**	**3h**	Tacrine
Molecular weight	360.45	374.48	388.51	402.53	416.56	430.59	444.61	458.64	200.28
No. of H-bond donors	2	2	2	2	2	2	2	2	2
No. of H-bond acceptors	5	5	5	5	5	5	5	5	2
No. of rotatable Bonds	5	6	7	8	9	10	11	12	0
TPSA (Å^2^)^a^	66.91	66.91	66.91	66.91	66.91	66.91	66.91	66.91	38.38
Fraction unbound in brain (fu, brain)^b^	0.05	0.04	0.02	0.01	0.01	0.01	0.01	0.01	0.15
Log BB^a^	**−**0.12	−0.06	0.29	0.49	0.55	0.50	0.55	0.44	0.21
Log PS^b^	**−**1.91	−1.84	**−**1.72	**−**1.63	**−**1.68	**−**1.94	**−**2.19	**−**2.49	**−**2.05
Log (PS*fu, brain)^b^	**−**3.22	−3.30	−3.40	**−**3.59	**−**3.77	**−**4.12	**−**4.38	**−**4.69	**−**2.87
Log *p*	3.49	3.75	4.18	4.79	5.24	5.88	6.29	6.72	2.60
Fraction unbound in plasma	0.037	0.031	0.041	0.034	0.029	0.021	0.023	0.017	0.24
Probability of positive Ames test^a^	0.59	0.41	0.54	0.57	0.56	0.50	0.53	0.41	0.77

a According to the classification made by Ma et al.: compounds with log BB more than 0.3 cross the BBB readily, compounds with log BB less than −1.0 are poorly distributed to the brain.

b Other estimated parameters related to brain penetration were used to classify the compounds as CNS permeable or non-permeable: rate of brain penetration (Log PS) is the rate of passive diffusion/permeability; brain/plasma equilibration rate (Log(PS*fu, brain)); fu, brain – fraction unbound in plasma.

## Conclusion

In conclusion, a series of cyclopentaquinoline hybrids were designed and synthesised as multifunctional ChEIs. The biological evaluation showed that all of the novel compounds were good AChE/BuChE inhibitors in the micromolar range. Especially, **3b** derivative showed the highest potent ChEs inhibitory activities which were 0.052 µM for AChE (3-times and 2-times more active than tacrine and donepezil, respectively) and 0.158 µM for BuChE (75-times more active than donepezil). Some compounds were highly selective for AChE (i.e. **3a**, **3b,** and **3e** derivatives) and for BuChE (i.e. **3f**, **3g,** and **3h** derivatives). Kinetic assays and molecular modelling studies proved mixed-type of inhibition for **3b** compound for AChE and BuChE. In addition, the most active inhibitor towards AChE exhibited significant inhibition of Aβ (1–42) aggregation (ranging from 25.69% at the concentration of 5 µM to 92.78% at 100 µM). Additionally, examined **3b** compound showed non cytotoxic properties (compared to the concentration of IC_50_) and good anti-hyaluronidase activity (anti-inflammatory effect). ADMET profiling of the compounds were done to obtain molecules with important pharmacokinetic and pharmacodynamics properties. **3b** compound which was selected for further research showed significant drug-like characteristics according to the Lipinski’s rule of five. All the presented studies promote **3b** compound as a novel multifunctional agent for the treatment of AD, as well as presented derivative might be considered for further research as a promising target.
