# Correction to: New cyclopentaquinoline hybrids with multifunctional capacities for the treatment of Alzheimer’s disease

**DOI:** 10.1080/14756366.2018.1446394

**Published:** 2018-03-13

Czarnecka K, Girek M, Maciejewska K, et al. New cyclopentaquinoline hybrids with multifunctional capacities for the treatment of Alzheimer’s disease. J Enzyme Inhib Med Chem 2018;33:158-170.

Several corrections of this article are necessary due to errors in the Methods and results sections describing the performed enzyme kinetic measurements.

1. In the “Materials and methods” section the two paragraphs *“Kinetic characterisation of AChE inhibition”* and *“Kinetic characterisation of BuChE inhibition”* on page 161 have to be replaced by the following paragraph.
  Characterisation of AChE and BuChE inhibition
  The inhibitory potency of compound **3b** against AChE and BuChE was further characterized in kinetic measurements using the Ellman’s method^1^^,^^2^ in a 96-well microplate reader at 412 nm with ATC iodide as substrate (0.05–0.50 mM in assay) in presence of 0.4 mg/ml DTNB (total assay volume 140 µl). The measurements were performed in triplicates over a period of 5 min with AChE (2 U/ml in assay) in presence of 0.11 µM, 0.22 µM, and 0.55 µM of inhibitor **3b**, and with BuChE (4 U/ml in assay) at inhibitor concentrations of 0.2 µM, 1.1 µM and 2.2 µM. The reciprocal activities were plotted as function of the reciprocal substrate concentrations at the indicated inhibitor concentrations in form of Lineweaver-Burk plots.
2. Tables 2 (page 164) and 3 (page 165) in the “Results and discussion” section have to deleted.
3. Furthermore, Figures 1 and 2 of the original manuscript have to be replaced by the revised Figures 1 and 2, which are provided below.

**Figure 1.**
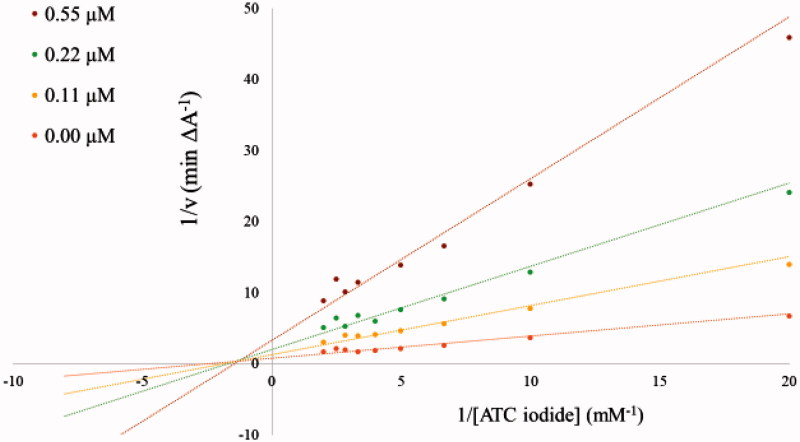
Lineweaver–Burk plots of the AChE inhibition by compound **3b** at different concentrations as indicated in the insert. All measurements were performed with 2 U/ml AChE at the shown substrate concentrations.

**Figure 2.**
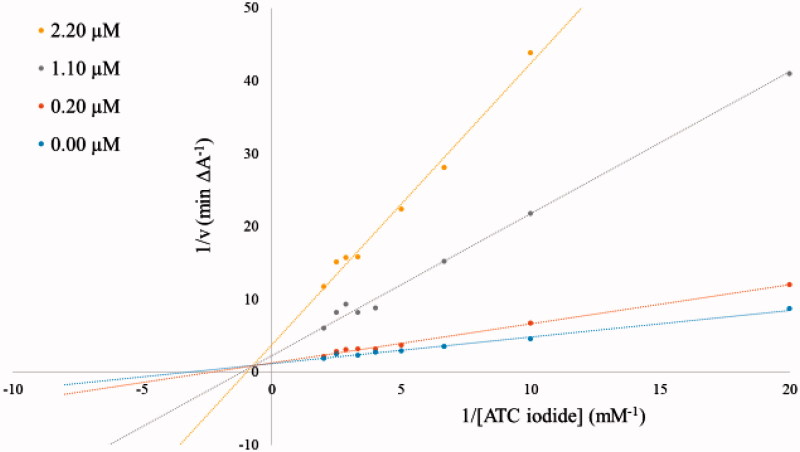
Lineweaver–Burk plots of the BuChE inhibition by compound **3b** at different concentrations as indicated in the insert. All measurements were performed with 4 U/ml BuChE at the shown substrate concentrations.
4. The text between page 163 (right column, starting with: *The mechanism of EeAChE inhibition* …) and page 164 (left column, ends with …Lineweaver-Burk plot revealed the mixed type of inhibition) has to be replaced by the following paragraph:

Due to the relatively strong inhibitory potency of compound **3b** against AChE (*IC*_50_ = 0.052 µM, Table 1) its potency was further investigated. The obtained Lineweaver–Burk plots for the inhibition of AChE (Figure 1) and BuChE (Figure 2) by compound **3b** revealed a deviation from a normal competitive inhibition mechanism and rather suggested a mixed-type of inhibition. A similar behaviour was recently described for a series of pyridine-derived AChE inhibitors. It was shown by docking studies that the inhibitor can interact with two different binding sites of AChE, the CAS (catalytic active site) and PAS (peripheral anionic site), which leads to a mixed-type of inhibition^3^.
